# Global DNA hypermethylation-associated cancer chemotherapy resistance and its reversion with the demethylating agent hydralazine

**DOI:** 10.1186/1479-5876-4-32

**Published:** 2006-08-07

**Authors:** Blanca Segura-Pacheco, Enrique Perez-Cardenas, Lucia Taja-Chayeb, Alma Chavez-Blanco, Alma Revilla-Vazquez, Luis Benitez-Bribiesca, Alfonso Duenas-González

**Affiliations:** 1 Unidad de Investigación Biomédica en Cáncer, Instituto de Investigaciones Biomédicas, Universidad Nacional Autonóma de Mexico, Instituto Nacional de Cancerología, Mexico; 2 Lab. de Desarrollo de Metodos Analiticos, FES-Cuautitlan, UNAM, Cuautitlan Izcalli, Estado de Mexico, Mexico; 3 Unidad de Investigacion Medica en Enfermedades Oncologicas, Hospital de Oncologia, CMN-SXXI, IMSS, DF, Mexico

## Abstract

**Background:**

The development of resistance to cytotoxic chemotherapy continues to be a major obstacle for successful anticancer therapy. It has been shown that cells exposed to toxic concentrations of commonly used cancer chemotherapy agents develop DNA hypermetylation. Hence, demethylating agents could play a role in overcoming drug resistance.

**Methods:**

MCF-7 cells were rendered adriamycin-resistant by weekly treatment with adriamycin. Wild-type and the resulting MCF-7/Adr cells were analyzed for global DNA methylation. DNA methyltransferase activity and DNA methyltransferase (*dnmt*) gene expression were also determined. MCF-7/Adr cells were then subjected to antisense targeting of *dnmt1, -3a*, and -*b *genes and to treatment with the DNA methylation inhibitor hydralazine to investigate whether DNA demethylation restores sensitivity to adriamycin.

**Results:**

MCF-7/Adr cells exhibited the multi-drug resistant phenotype as demonstrated by adriamycin resistance, *mdr1 *gene over-expression, decreased intracellular accumulation of adriamycin, and cross-resistance to paclitaxel. The mdr phenotype was accompanied by global DNA hypermetylation, over-expression of *dnmt *genes, and increased DNA methyltransferase activity as compared with wild-type MCF-7 cells. DNA demethylation through antisense targeting of *dnmts *or hydralazine restored adriamycin sensitivity of MCF-7/Adr cells to a greater extent than verapamil, a known inhibitor of mdr protein, suggesting that DNA demethylation interferes with the epigenetic reprogramming that participates in the drug-resistant phenotype.

**Conclusion:**

We provide evidence that DNA hypermethylation is at least partly responsible for development of the multidrug-resistant phenotype in the MCF-7/Adr model and that hydralazine, a known DNA demethylating agent, can revert the resistant phenotype.

## Background

Over the last several years, improved cancer therapies have permitted small gains in cancer survival. Currently, 5-year relative survival rates from all sites stand at 60% [1]. Remarkable advances in understanding neoplastic progression at cellular and molecular levels have spurred the discovery of molecularly targeted drugs [2]. Nevertheless, at present, conventional cytotoxic chemotherapy is still used in the majority of patients with cancer who require systemic treatment. Thus, it is vital to continue efforts in the study of mechanisms of chemotherapy resistance to seek means for its reversal.

While gain, loss, and mutation of genetic information have long been known to contribute to cancer development and progression, it is being increasingly recognized that epigenetic defects may play an equally important role. The pathological epigenetic changes that contribute to cancer development include global DNA hypomethylation, hypermethylation of specific genes, chromatin remodeling and loss of imprinting [3]. Loss of genomic methylation is a frequent and early event in cancer, and correlates with disease severity and metastatic potential in many tumour types [3]. But it is generally accepted that one of the main epigenetic modifications of the human genome is cytosine residue methylation within the context of the CpG dinucleotide as executed by at least three functional DNA methyltransferases: dnmt1, which preferentially methylates hemi-methylated DNA and plays a key role in imprinting and X-chromosome inactivation during embryogenesis [4], and *de novo *methyltransferases, dnmt3a and dnmt3b responsible for *de novo *methylation during embryogenesis and which possess equal preference for hemi- and non-methylated DNA and that therefore have been classified as *de novo *methyltransferases [5,6]. DNA methylation can directly interfere with the binding of transcription factors to inhibit replication [7] and/or methyl-CpG binding proteins that can bind to methylated DNA, as well as regulatory proteins to inhibit transcription [8]. In addition, both *dnmt1 *and methyl binding proteins such as methyl-CpG-binding protein 2 (MeCP2) recruit histone deacetylases that by deacetylation of core histone tails lead to tighter packing of DNA into chromatin, reducing access of transcription factors [9,10].

*De novo *methylation of CpG islands in tumor suppressor gene promoter regions may lead to transcriptional silencing through a complex process involving histone deacetylation and chromatin condensation, and thus represents a tumorigenic event that is functionally equivalent to genetic changes such as mutation and deletion [11]. Therefore, better understanding of epigenetic mechanisms leading to tumor formation and chemoresistance may eventually improve current cancer treatment regimens and instructive for more rational use of anticancer agents.

It has long been recognized that cultured cells exposed to a variety of commonly used cancer chemotherapy agents, particularly at high concentrations, develop DNA hypermethylation; hence, drug-induced DNA hypermethylation is thought to constitute one component of human tumor cell response to toxic concentrations of commonly used cancer chemotherapy agents. Thus, drug-induced DNA hypermethylation may be capable of creating drug-resistant phenotypes by inactivating genes whose products are required for drug cytotoxicity [12]. In this work through use of an MCF-7/Adr-resistant model, we demonstrate that DNA hypermethylation leads to drug resistance that can be reverted by either down-regulating DNA methyltranferase genes by antisense oligonucleotides or by pharmacologic reversion of methylation by hydralazine, a known DNA methylation inhibitor [13-16].

## Methods

### Cell culture

MCF-7 human breast adenocarcinoma cells were cultured at 37°C in a humidified atmosphere containing 5% CO_2 _in DMEM supplemented with 10% (v/v) fetal calf serum and sub-cultured using 0.25% trypsin with 1 mM EDTA (Life Technologies, Inc.,). Adriamycin-resistant MCF-7 cells (MCF-7/Adr) were established in our laboratory by intermittent exposure to adriamycin at 100 ng/mL for 1 h every week for 20 weeks. Afterward, MCF-7/Adr cells were routinely maintained in 200 ng/mL of adriamycin.

### Cytotoxicity assays

Cells were seeded into 96-well microtiter Falcon plates (Becton Dickinson, Franklin Lakes, NJ, USA) at 1.5–2.5 × 10^3 ^cells/well in 0.1 mL of complete medium. The following day, cells were treated with adriamycin, paclitaxel, cisplatin, or 5-fluorouracil for 24 h, and subsequently cell viability was measured by MTT dye reduction assay. Experiments for resistance reversion were performed as follows: Cells were cultured as previously described and were pre-treated for 4 days with 10 μM hydralazine or for 24 h with verapamil at 5 μM. Following this, the medium was replaced with one containing adriamycin to then evaluate viability 24 h later. Briefly, 50 μL of 5 mg/mL MTT reagent in PBS were added to each well. Viable cells with active mitochondria reduce the MTT to an insoluble purple formazan precipitate that is solubilized by the subsequent addition of 150 μL of DMSO. The formazan dye was measured spectrophotometrically using an ELISA reader. All assays were performed in triplicate. The cytotoxic effect of each treatment was expressed as a percentage of cell viability relative to untreated control cells (percentage of control) and is defined as [(*A*_570 nm-_treated cells)/*A*_570 nm-_nontreated cells)] × 100.

### Nucleic acid extraction from cells

Genomic DNA was obtained with the standard method of proteinase-K digestion and phenol-chloroform extraction. RNA was obtained using the Trizol Reagent (Gibco BRL, Grand Island, NY, USA) RNA extraction kit following manufacturer instructions.

### DNA methylation assays

Capillary electrophoresis. Quantification genomic 5-methylcytosine DNA content by capillary electrophoresis was performed as previously described [17]. Briefly, DNA hydrolysis was carried out by incubating 40 μg of DNA in 2 mL of 88% v/v formic acid at 140°C in a sealed ampoule for 90 min. After hydrolysis, samples were reduced to dryness by speedvac concentration and redissolved in 30 μL of Milli-Q-grade water. An uncoated fused-silica capillary (60 cm × 75 μm; effective length, 44.5 cm) (Beckman-Coulter was used in a CE system (P/ACE MDQ, Beckman-Coulter) connected to a 32 Karat software data-processing station. The running buffer was 20 mM NaCO_3 _(pH 9.6 ± 1) containing 80 mM SDS. Running conditions were 25°C with an operating voltage of 20 kV. On-column absorbance was monitored at 223 nm. Prior to each run, the capillary system was conditioned by washing with the running buffer for 2 min. Hydrolyzed samples previously filtered through 0.45-μm pore filters were injected under pressure (0.5 p.s.i.) for 15 sec. Results were expressed as absolute percentage of cytosine and 5-methylcytosine and assays were performed in triplicate.

Cytosine-extension assay. DNA methylation was also determined by this assay essentially as described [18]. This assay is based on use of methylation-sensitive restriction endonuclease that leaves a 5'guanine overhang after DNA cleavage, with subsequent single nucleotide extension with radiolabeled [(3)H]dCTP. Briefly, 1 μg of DNA from cells was digested overnight with HpaII or MspI according to manufacturer instructions. Single nucleotide extension reaction was performed in a 25-μL reaction mixture containing 0.25 μg of DNA, 1X buffer II, 1 mM MgCl, and 0.25 U of DNA polymerase [^3^H]dCTP (Ci/mmol) (Perkin-Elmer, Branchburg, NJ,. USA), incubated at 56°C for 1 h, and subsequently placed on ice. The reaction mixture was then applied to Sephadex G25 column. For column chromatography, each Sephadex G25 column was centrifuged for 10 sec at 5,000 rpm to remove the buffer, and then loaded with the reaction mixture. After loading samples onto the column, radiolabeled DNA was collected by column centrifugation and mixed with liquid scintillation for determination of radioactivity. Assays were done by triplicate and results were expressed as percentage change from average controls

Dot blot assay. A total of 5 μg of DNA previously digested with EcoRI for 1 h was dot-blotted onto a nitrocellulose membrane (Bio-Rad) in a total volume of 2 μL per sample. Membranes were allowed to dry and then UV-crosslinked for 15 sec. Membrane blocking was done by soaking in 2.5% non-fat milk in TBS (1 h at room temperature) prior to incubation with a primary sheep polyclonal antibody (1:200 dilution dissolved in 2.5% TBS-T overnight at 4°C) against 5-methylcytosine (Maine biothecnology Services INC, Portland). Membranes were washed three times with TBS-T, 5 min each and then incubated with secondary antibody conjugated with HRP at 1:1000 dilution (Santa Cruz) for 30 min at RT, washed with TBS-T (once for 15 minm twice for 5 min and then once with TBS for 5 min. Membranes were incubated with ECL reagent for 1 min and expose X-ray film in the dark room. Signal intensity was quantified by densitometry. Assays were done by triplicate.

### DNA methyltransferase assay

The *in vitro *methylation assay was performed as follows: total cell extract was obtained with lysis buffer (50 mM Tris-HCl pH 8.0, EDTA 1 mM, 0.001% Na azide, 10 % glycerol, 1 mM DTT, 0.06 mg/mL) (Sigma,). DNA methyltransferase activity was measured employing 500 ng of a 1,112-bp fragment of the type-I Herpes simplex virus tymidine kinase gene, which has a high GC content, in presence of 50 mg cellular protein obtained from MCF-7 or -7/Adr cells previously exposed to 0–20 μM hydralazine for 5 days. Final reaction volume (50 μL) was completed with 3 μL of S-adenosyl-L- [methyl-^3^H]methionine (1 mCi/mL) (Amersham, Buckinghamshire, UK). Reactions were conducted at 37°C for 2 h and stopped with 350 μL of stop solution (1% SDS, 2 mM EDTA, 0.04 g/mL 4-aminosalicylate, 125 mM NaCl, 0.25 mg/mL salmon testis DNA, 1 mg/mL proteinase K). DNA was purified using phenol-chloroform and precipitated with cold ethanol. DNA was resuspended in 30 μL of 0.3 M NaOH and spotted on Whatman DE81 filter paper disc (Whatman, Maidstone, UK). The disc was washed three times in 5% trichloroacetic acid (J.T. Baker, Xalostoc, Edo. de México. Mexico) with BSA (Research Organics, Cleveland, OH, USA) and three times in 70% ethanol, and dried. Radioactivity was measured in a Beckman liquid scintillation counter (LS 6000TA). A blank control reaction was carried out simultaneously using only lysis buffer. The results, expressed in dpm, were adjusted by subtracting the background level. Each assay was performed in triplicate.

### RT-PCR

Total RNA was reverse transcribed using a RT-PCR kit (Perkin Elmer) following manufacturer instructions. Primers and conditions for amplifications were as follows: *dnmt1*, sense AGCCTTCGGCTGACTGGCTGG antisense CTGCCCATCATCATGACCTGG product size 150 bp, annealing 60°C. *dnmt3a*, sense GACTGTATGGATGTTCTGTCAG antisense ATTTGTCCTGGCAGACGAAGCA product size 146 bp, annealing 50°C. dnmt3b, sense GCTGCAGACCAVCTCTGTGGCACG antisense GCCGCCTCTTCACCATCCCG product size 81 bp, annealing 50°C. *mdr1*, sense ATGTCGTTCAGATTTGGCCAAC antisense TCATAGATGCTGTCATTCCTGT product size 340 bp, annealing 53°C.

### Adriamycin accumulation by flow cytometry

Adriamycin is a small heterocyclic amine (molecular mass, 580 daltons) with a pK_a _of 8.3 that can diffuse across membranes in the uncharged form. Adriamycin fluorescence is excited between 350 and 550 nm and emits between 400 and 700 nm. MCF-7 and -MCF-7/Adr cells were cultured in flasks and then treated for 1 h with adriamicyn at 3 μM. Afterward, cells were harvested with 0.25% trypsin and 1 mM EDTA and centrifuged. Cell pellets were washed twice with cold PBS, resuspended in 0.3 mL of PBS, and transferred to 352063 Falcon tubes. After 30 min, cell fluorescence intensity (10^4^/mL) was determined by flow cytometry using a FAC-Scalibar (Becton Dickinson, San Jose, CA, USA).

### Antisense treatment against Dnmts

MCF-7/Adr cells were transfected with the following phosphorothiorate antisense oligonucleotides as previously described [19]: *DNMT1*-AS, 5'-AAGCATGAGCACCGTTCTCC-3'; *DNMT1*-MM (mismatch), 5'-AACGATCAGGACCCTTGTCC-3'; *DNMT3A*-AS, 5'-CAGGAGATGATGTCCAACCC-3'; *DNMT3A*-MM, 5'CA CGACATCATCTCGAACGC-3'; *DNMT3B*-AS, 5'-CGTCGTGGCTCCAGTTACAA-3', and *DNMT3B*-MM, 5'CCTCGTCGGTCGACTTAGAA-3'. Over the course of 72 h, cells were transfected with 75 nM of each antisense oligonucleotide every 24 h (three treatments) with 6.25 μg/mL of lipofectine. Surviving cells were analyzed for viability and DNA methylation 24 h after the last transfection. Assays were done by triplicate.

## Results

### Generation of multi-drug resistance phenotype

**Figure 1:**
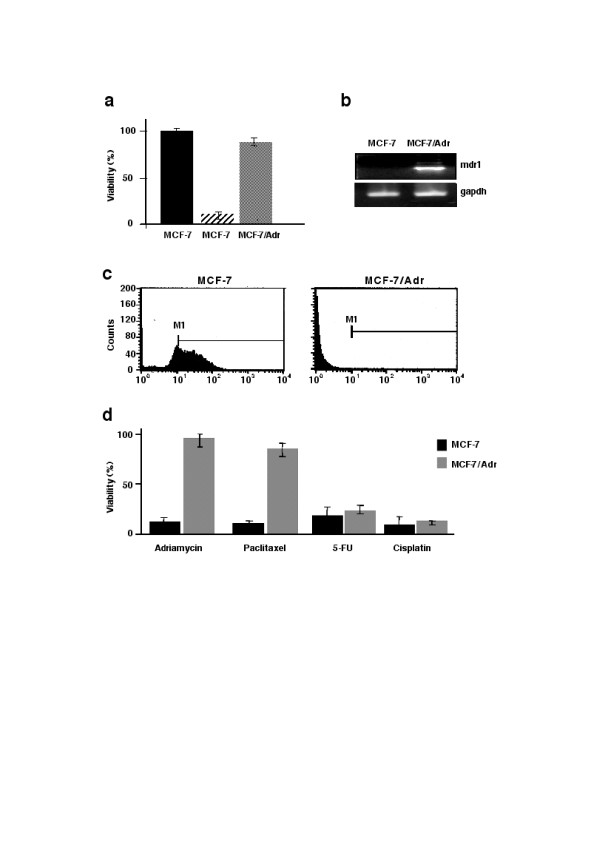
**MCF-7/Adr cells show the *mdr *phenotype**. a: Wild-type MCF-7 cells exposed to IC90 of adriamycin show expected growth inhibition, whereas MCF-7/Adr cells are resistant to adriamycin as evaluated by the MTT assay; b: RT-PCR analysis of the *mdr1 *gene demonstrates that MCF-7/Adr cells over-express the *mdr1 *transcript; c: Flow cytometric analysis of adriamycin-treated cells for 1 h show that wild-type MCF-7 cells retain the majority of adriamycin inside the cell (M1); however, MCF-7/Adr cells have essentially no drug accumulation, indicative of the presence of a functional mdr1 protein, and d: MCF-7/adr cells demonstrate cross-resistance to paclitaxel but not to 5-fluorouracil and cisplatin.

As expected, MCF-7 cells receiving weekly pulses of adriamycin acquired the resistant phenotype. MCF-7/Adr became resistant to adriamycin, exhibited over-expression of the *mdr1 *gene, and diminished intracellular accumulation of adriamycin as evaluated by flow cytometry. In addition, MCF-7/Adr demonstrated cross-resistance to paclitaxel but not to 5-fluorouracil and cisplatin (Figures 1a, b, c, and 1d).

### Global DNA hypermethylation is associated with mdr-phenotype

**Figure 2:**
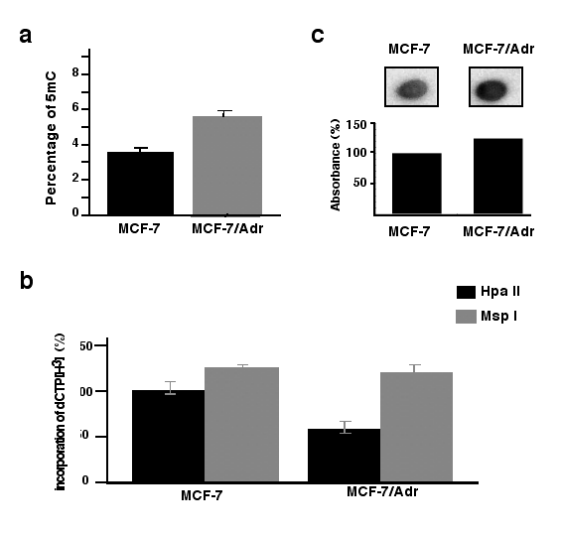
**Global hypermethylation in MCF-7/Adr cells**. a) 5-mC content was evaluated by capillary electrophoresis. Percentage of 5-mC was 3.6% in MCF-7, while this increased to 5.6% in MCF-7/Adr cells; b) cytosine-extension assay based on use of methylation-sensitive restriction endonuclease that leaves a 5' guanine overhang after DNA cleavage with subsequent single-nucleotide extension with radiolabeled [(3)H]dCTP shows a 40% decrease in incorporation of radiolabeled [(3)H]dCTP by the DNA of HpaII-digested MCF-7/Adr cells, indicative of hypermethylation, and c), a polyclonal antibody against 5-methylcytosine was incubated with the digested DNA spotted in a nitrocellulose membrane. Spot intensity is 30% higher as evaluated by densitometric analysis in the DNA of MCF-7/Adr cells, also indicative of DNA hypermethylation.

To evaluate whether or not DNA hypermethylation is associated with drug resistance acquisition, global DNA methylation was evaluated in MCF-7 and MCF-7/Adr. As shown in Figure 2a, there was an increase of nearly 2% in the absolute percentage of 5-methylcytosine (5-^m^C) content as evaluated by capillary electrophoresis. This observation was also corroborated by two additional methods: cytosine-extension assay (Figure 2b), and dot blot assay (Figure 2c).

### The mdr-phenotype is associated with over-expression of DNA methyltransferases

**Figure 3:**
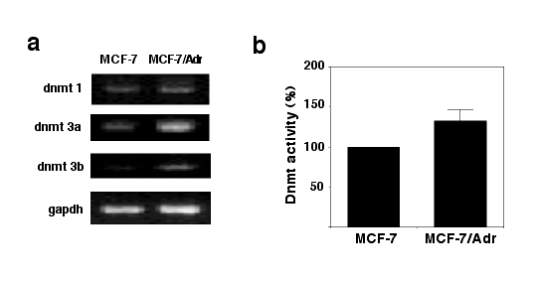
**Expression of DNA methyltransferase genes and DNA methyltransferase activity**. a) MCF-7 and MCF-7/Adr cells were growth-arrested and *dnmts *analyzed by RT-PCR. Wild- type cells had very low or no expression of *dnmt1*, *-3a*, and *-b *as compared with MCF-7/Adr cells; b) corresponding DNA methyltransferase activity was increased by 30% in resistant cells.

The presence of global and gene promoter-specific DNA hypermethylation in MCF-7/Adr-resistant cells suggested changes in the methylation machinery. Thus, we analyzed *dnmt *gene expression as well as DNA methyltransferase enzymatic activity of MCF-7/Adr in comparison with wild-type cells. Because it is known that expression of *dnmts *changes according to cycling status, cells were growth-arrested by serum deprivation for 24 h (corroborated by flow cytometry, not shown), and then gene expression was analyzed. Results showed clear over-expression of *dnmt1*, *-3a*, and *-b *in resistant cells as compared with MCF-7 cells (Figure 3a). Corresponding with these findings, DNA methyltransferase enzymatic activity was 40% higher in MCF-7/Adr cells (Figure 3b).

### Effects of DNA methyltransferase antisense treatment on drug-induced hypermethylation and chemotherapy resistance

**Figure 4:**
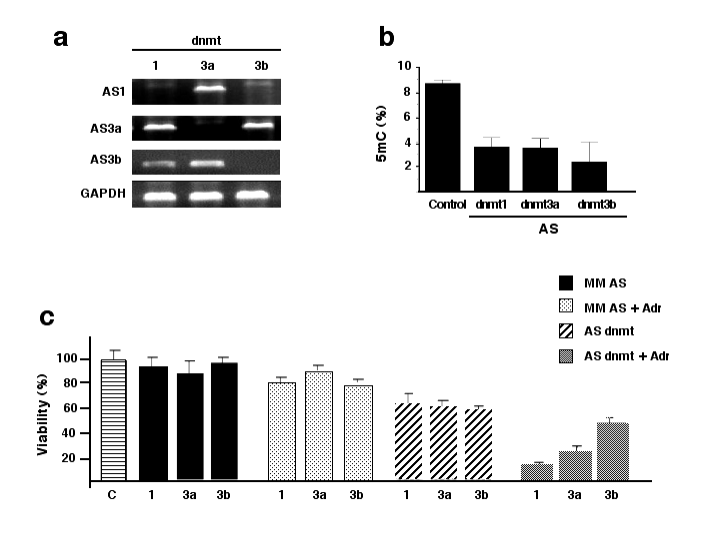
**Effects of DNA methyltransferase antisense treatment on drug-induced hypermethylation and chemotherapy resistance**. a) MCF-7/Adr cells had no expression of *dnmts *transcripts after 72 h of antisense treatments; b) transfected cells with the antisense oligonucleotides had a 5-mC content between 2 and 4%, as compared with 5.6% in untreated MCF-7Adr cells, and c) MTT assays demonstrate that cells transfected with mismatch (MM) oligonucleotides showed no reduced cell viability and only a small reduction when challenged with adriamycin. The sole transfection with oligonucleotides against *dnmts *led to a reduction in viability between 30 and 40%, and when treated with adriamycin strong cytotoxicity was observed being higher for *dnmt1 *followed by *-3a *and *-b*.

Despite the clear association between development of drug resistance and DNA hypermethylation, it remains unclear whether DNA hypermethylation contributes to the development of drug resistance or whether it is solely a characteristic of the resistant phenotype. With this aim, MCF-7/Adr cells were transfected with antisense oligonucleotides against each of the *dnmt *genes. After 72 h of antisense treatment, once lack or a strong diminishing of respective transcripts was confirmed in cells (Figure 4a), global DNA methylation was assessed, as well as viability with and without adriamycin treatment. Results indicate that while treated cells with mismatch oligonucleotides exhibited no growth inhibition with or without adriamycin, the sole knocking out of *dnmts *in MCF-7/Adr cells led to a small growth inhibition that, nonetheless, increased when these cells were treated with adriamycin, as shown in Figure 4c. Interestingly, adriamycin sensitivity was more evident in *dnmt1*-, and less evident in *-b*, antisense-treated cells. Sensitivity to adriamycin by means of antisense treatment correlated with decreased global methylation (Figure 4b).

### Drug resistance and DNA hypermethylation are reversed with hydralazine

**Figure 5:**
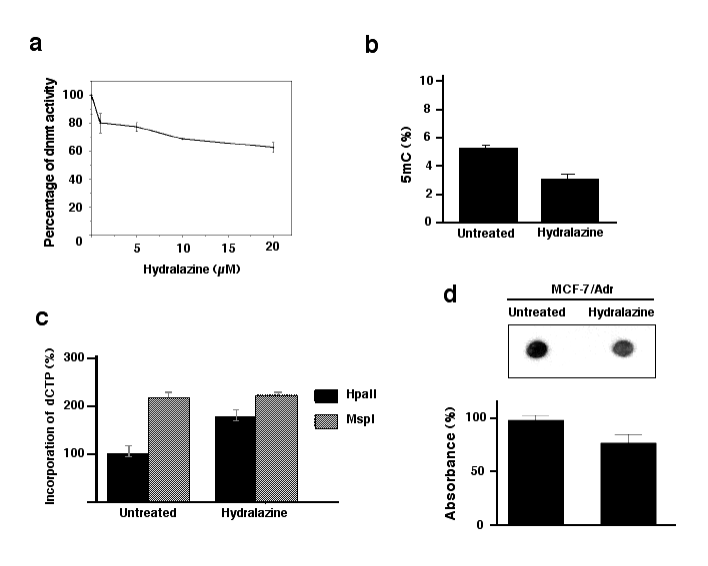
**Effects of hydralazine on DNA methylation and DNA methyltransferase activity**. a) MCF-7/Adr cells were treated with increasing concentrations of hydralazine for 4 days and then DNA methyltransferase activity assayed. Hydralazine at 10 μM decreased enzymatic activity by 30%; b) 5-mC content was also reduced by hydralazine from 5.6–2.7%; c) cytosine-extension assay also showed higher incorporation into hydralazine treated cells as reflected by the bar marked HpaII, as did (d), dot-blot evaluation of hydralazine-treated MCF7/Adr cells.

**Figure 6:**
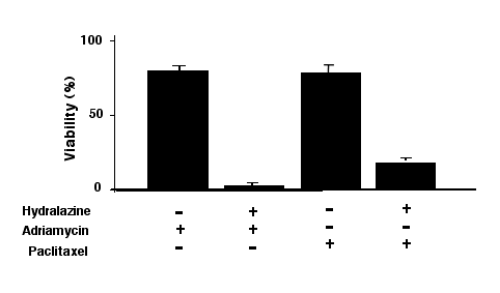
**Drug resistance and DNA hypermethylation are reversed with hydralazine**. MCF-7/Adr cell viability was evaluated with the MTT assay after treatment with adriamycin and paclitaxel. Cells pretreated with hydralazine had their sensitivity to these drugs restored.

Because MCF-7/Adr antisense treatment not only leads to DNA demethylation but also to reversal of resistance, we sought to determine whether the demethylating agent hydralazine could also be able to restore sensitivity to adriamycin. First, we measured the DNA methyltransferase activity in MCF-7/Adr cells after hydralazine treatment. Results demonstrate that hydralazine induces dose-dependent decreases in DNA methyltransferase enzymatic activity, a 30% decrease at 10 μM, the dose used for demethylation in cell culture experiments (Figure 5a), and that it also reduces the global DNA methylation level to that observed in wild-type MCF-7 cells (Figures 5b,5c,5d). As expected, cell viability experiments showed that hydralazine rendered MCF-7/Adr cells sensitive to adriamycin at a level indistinguishable to that of wild-type MCF-7. This restoration of sensitivity by hydralazine was also observed when cells were treated with paclitaxel, on which adriamycin-resistant cells showed cross-resistance (Figure 6).

### The *mdr *fphenotype of MCF-7/Adr cells and its reversion with hydralazine and verapamil

**Figure 7:**
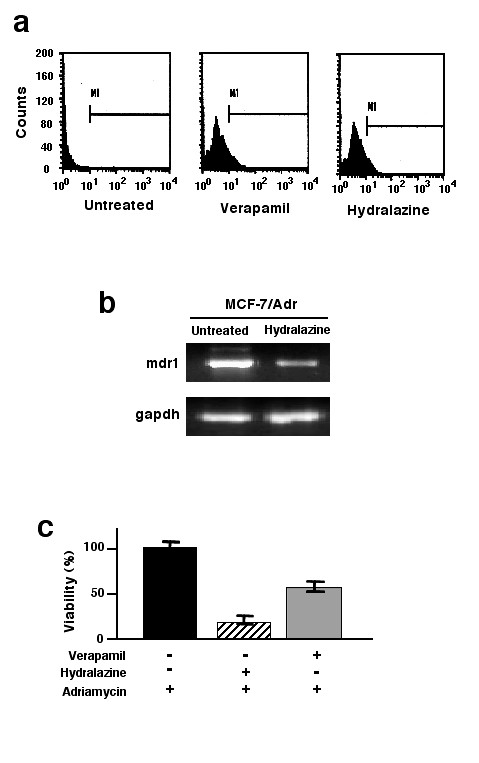
**The mdr phenotype of MCF-7/Adr cells and its reversion with hydralazine and verapamil**. a) Untreated MCF-7/Adr cells had no intracellular retention of adriamycin as indicated by MI, whereas verapamil and hydralazine clearly led to drug retention, and b) hydralazine decreased *mdr1 *gene expression as evaluated by RT-PCR. Figure 7c demonstrates that pre-treatment with verapamil led to only a 50% decrease in viability when cells were exposed to adriamycin as compared with an 85% reduction achieved with hydralazine pre-treatment.

It is widely known that the MCF-7/Adr model expresses the mdr-phenotype characterized by *mdr1 *gene and protein over-expression. To gain further insight into the role that *mdr *plays in comparison with DNA hypermethylation for chemoresistance in this model, we decided to determine to what extent verapamil, a known allosteric inhibitor of the mdr protein, could reverse adriamycin resistance in MCF-7/Adr cells. As shown in Figure 7a, adriamycin accumulation in MCF-7/Adr cells was almost none. As expected, resistant verapamil-treated cells experienced an increase in intracellular adriamycin accumulation comparable to that with hydralazine. As there is no data to suggest direct interaction of hydralazine with mdr protein, we sought to determine whether hydralazine by its demethylating effect could inhibit *mdr1 *gene expression. Results demonstrate that hydralazine decreases rather than increases expression of the *mdr1 *(Figure 7b). Our data demonstrates that hydralazine by its effect on *mdr1 *gene expression increased cellular accumulation of adriamycin to the same extent as verapamil; hence, we decided to compare the individual ability of these compounds to reverse resistance to adriamycin. Interestingly, the results showed that despite that both drugs led to comparable accumulation of adriamycin, hydralazine rendered MCF-7/Adr more susceptible to adriamycin than verapamil (Figure 7c).

## Discussion

Earlier studies underscored that generation of a drug-resistant phenotype was accompanied by DNA hypermetylation; nevertheless, no causal relationship between these could be established [20,21]. Results of the present work performed in a well-established model of chemoresistance, the MCF-7/Adr cells, demonstrate that cells require DNA hypermetylation to acquire the mdr phenotype, and that once cells become resistant their sensitivity can be restored by treatment with either a DNA methylation inhibitor or by down-regulation of *dnmt*s with antisense oligonucleotides.

The phenotype of MCF-7/Adr cells includes over-expression of a functional mdr, diminished intracellular accumulation of adriamycin, and cross-resistance to other agents such as paclitaxel [22]. Herein, we demonstrate that global DNA hypermethylation is also a feature of this model by use of capillary electrophoresis, cytosine-extension assay, and dot blot. Whether this hypermethylation has predilection over specific regions of the genome remains to be established. This increased methylation results from *dnmt*s over-expression and increased DNA methylation enzymatic activity, as shown in Figure 3, in which we demonstrate a clear increase in expression level and activity in resistant cells. The exact mechanism by which cells over-express *dnmt*s after drug exposure remains to be determined however, it is likely that it results from chemotherapy-induced phosphorylation/activation of mitogen-activated protein (MAP) kinases, such as extracellular signal-regulated kinase (ERK) 1/2 [23] which is known to participate in the regulation of dnmts gene expresión [24]. DNA hypermethylation has also been observed in drug-resistant murine neuroblastoma cells that exhibit over-expression of *dnmt1 *and *-3b *[25]. These data suggest that *dnmt *over-expression may aid in stress-induced epigenetic cell reprogramming as an important prerequisite to survival [26].

The association between chemoresistance and global hypermetylation is well established; accordingly, antisense experiments demonstrated that knocking out either one of the three *dnmts *reduced the 5^m^C content to different extents and exhibited a discrete growth-inhibitory effect in MCF-7/Adr cells, suggesting that changes in methylation are crucial for these cells to maintain their growth. Furthermore, exposure of antisense-treated cells to adriamycin led to nearly complete restoration of sensitivity to the drug. The effect upon cell viability among the three antisense-treated cells was roughly the same; nonetheless, *dnmt1 *antisense-treated adriamycin cells showed greater sensitivity restoration, suggesting that this enzyme could play the major role in drug resistance acquisition in this model. The precise role for the different *dnmts *in cancer development and progression remains under study; however, it is known that there is ample variability in the level of expression of these genes in a number of tumors. For instances, in a study of the expression level of all three methyltransferase genes by RT-PCR from bladder, colon, kidney, and pancreas tumors, mean fold increases were 4, 3.1, and 7.5 for *dnmt1*, *-3a*, and *-b*, respectively [27]. The same pattern of expression degree was observed in acute myelogenous leukemia (higher for *dnmt3b*, followed by *-1 *and then *-3a*) [28] and breast carcinomas [29]; nevertheless, in the acute phase of chronic leukemia *dnmt3a *predominates over *-3b *and *-1 *[28], whereas in acute lymphoblastic leukemia *dnmt1 *expression level was higher than *-3a *and *-b *[30]. Thus, our finding of higher restoration of adriamycin sensitivity in *dnmt1*-depleted cells would suggest that in the MCF-7/Adr model *dnmt1 *exhibits the higher participation. Recently, it was shown that in genetically modified human cancer cells including *dnmt3a- *and *-b*-deficient cells, *dnmt1 *is the major *de novo *methyltransferase for three different CpG island substrates, suggesting that *dnmt1 *might be considered to possess more diverse and wide-ranging catalytic activities than previously suspected for a simple maintenance enzyme [31]. Nevertheless, the contribution of *dnmt3a *and *-b *enzymes for the resistant phenotype cannot be neglected. A recent study in murine neuroblastoma cells demonstrates that over-expression of *dnmt3a *and *-b *by transfection leads to cisplatin resistance, which can be reverted by treating these cells with 5-azacytidine [32]. Thus, our results and those from the neuroblastoma model clearly indicate that DNA methylation plays a central role in onset of drug resistance.

Because of the demonstrated participation of hypermethylation for development of resistance in this model, we wanted to determine whether the non-nucleoside inhibitor of DNA methylation hydralazine [13-16] could also restore adriamycin sensitivity. Our results demonstrate that hydralazine decreases DNA methyltransferase activity and methylation to levels shown in wild-type cells, this as expected due to its methyltransferase inhibitory activity [16] as well as due to its ability to decrease *dnmt1 *and *-3a *gene expression [16,33].

Hydralazine is a demethylating agent that has demonstrated the ability to down- and up-regulate a number of genes [34] and as such, not only affects *mdr1 *status in our model but also potentially other genes that could be implicated in developing drug resistance. Thus, to evaluate whether the hydralazine's reversing effect upon resistance to adriamycin depends on only *mdr1 *or on other transcriptional changes we compared it against verapamil, an allosteric inhibitor of the *mdr *pump [35]. Interestingly, despite that hydralazine produces a comparable effect to that of verapamil upon adriamycin accumulation (Figure 7a), its effects were higher than those of verapamil in cytotoxicity assays, suggesting that its epigenetic actions reactivate or down-regulate additional genes that according to the epigenetic reprogramming hypothesis participate in the drug-resistant phenotype. An issue not addressed in our work was the mechanism underlying the observed expression change at *mdr1 *gene. The precise mechanism of transcriptional regulation of this gene has remained unclear due to its complex regulatory nature, as a consequence, studies performed in both cancer cell lines and clinical samples have shown that *mdr1 *promoter methylation density either inversely [36-41] or directly [42][43][44][45,46], correlates with *mdr1 *gene expression. Whatever the mechanism, it is now clear that increased drug efflux potential by ABC-transporters such as mdr1 is only one of the multiple polygenic pathways characterizing the drug resistant state [47].

In conclusion, the results of this study in the MCF-7/Adr model demonstrate that global DNA hypermethylation participates in development of adriamycin resistance and that the demethylating agent hydralazine can revert the resistant phenotype. In this sense it has been recently hypothetized that epigenetic reprogramming can participate in the establishment of an epigenetic mark associated with the chemotherapy resistant phenotype and that DNA methylation and histone deacetylation inhibitors can restore chemotherapy sensitivity [48]. We are currenty evaluating in phase II studies the ability of hydralazine plus magnesium valproate, a histone deactylase inhibitor to resensitize tumor cells to chemotherapy in refractory solid tumors as well as their ability to improve tumor response when these epigenetic agents are added to adriamycin-based neoadjuvant therapy in locally advanced breast cancer.

## Competing interests

The author(s) declare that they have no competing interests.

## Authors' contributions

B S-P performed the majority of the experimental work; L T-C, E P-C, and A C-B contributed with part of the experimental work; AR-V participated in the methylation analysis; L B-B critically read and contributed to the manuscript, and A D-G conceived of and wrote the manuscript. All co-authors critically read and approved the manuscript.
